# Vitalism and cognition in a conscious universe

**DOI:** 10.1080/19420889.2022.2071102

**Published:** 2022-05-08

**Authors:** Marco Masi

**Affiliations:** Independent Scholar, Germany

**Keywords:** Vitalism, basal cognition, mind, consciousness, morphogenesis, synthetic biology, philosophy, philosophical idealism

## Abstract

According to the current scientific paradigm, what we call ‘life’, ‘mind’, and ‘consciousness’ are considered epiphenomenal occurrences, or emergent properties or functions of matter and energy. Science does not associate these with an inherent and distinct existence beyond a materialistic/energetic conception. ‘Life’ is a word pointing at cellular and multicellular processes forming organisms capable of specific functions and skills. ‘Mind’ is a cognitive ability emerging from a matrix of complex interactions of neuronal processes, while ‘consciousness’ is an even more elusive concept, deemed a subjective epiphenomenon of brain activity. Historically, however, this has not always been the case, even in the scientific and academic context. Several prominent figures took vitalism seriously, while some schools of Western philosophical idealism and Eastern traditions promoted conceptions in which reality is reducible to mind or consciousness rather than matter. We will argue that current biological sciences did not falsify these alternative paradigms and that some forms of vitalism could be linked to some forms of idealism if we posit life and cognition as two distinct aspects of consciousness preeminent over matter. However, we will not argue in favor of vitalistic and idealistic conceptions. Rather, contrary to a physicalist doctrine, these were and remain coherent worldviews and cannot be ruled out by modern science.

## I. Introduction

From the perspective of scientific materialism, it is generally believed that metaphysical vitalism (that is, the hypothesis of an origin and life theory depending on a force or principle distinct from chemical or physical forces) or physical vitalism (a non-reductionist understanding of life which claims that an organism with its generative, developmental and living functions cannot be reduced to the sum of its parts) must both be relegated to a status as relics from the history of science [1]. Especially metaphysical vitalism was criticized beginning with the rise of positivism. Philosophers of science since the Vienna circle [2,3**]**, and later mid-20^th^ century empiricists Ernest Nagel [4], and Gustav Hempel [5], have rejected neovitalist tendencies.

This exclusion is because vitalist conceptions are generally at odds with a scientific reductionist and physicalist approach while being more congruent with Western theories of philosophical idealism or Eastern philosophies.

Physicalism, also called ‘material monism’ or just ‘materialism’,^a^ assumes that everything can be reduced to material constituents and that the physical world can be explained by physical laws without any reference to metaphysical concepts. Physical matter is the fundamental reality and current paradigm of scientific materialism used to define ‘life’, ‘mind’, and ‘consciousness’.

On the other hand, philosophical idealism represents an alternative paradigm of reality. We will focus on a particular version of idealism, so-called ‘objective’ or ‘ontological’ idealism,^b^ and call it just idealism. Idealism is a metaphysical perspective according to which reality cannot be reduced to matter but is itself fundamentally of a mental cognitive nature. It posits mind as a basic primitive of reality–that is, as something that cannot be defined in terms of previously-defined concepts. The Western conception of the word ‘mind’ sometimes includes ‘consciousness’, ‘spirit’, or ‘will’.^c^ Idealism postulates that all of reality is a form of conscious thought and cognition that exists independently of human thought and is prior to conscious life. From this idealist perspective, life, mind, and consciousness are not conceived as biological emergent phenomena or properties of matter, as physicalism posits. Rather, to the contrary, life and matter are considered to emerge from the only mental ‘one-substance’ (mind, consciousness, will, or spirit). Idealist philosophers, like Baruch Spinoza, Arthur Schopenhauer, George Berkeley, Friedrich Hegel, and Friedrich Schelling, to mention just some, replaced material monism (“there is only matter”) with a ‘mental-’ or ‘substance-monism’ (“there is only mind”).

Eastern philosophies offer a different approach. In particular, the non-dual Advaita Vedanta (with many similarities to Buddhism and other Indian doctrines) posits consciousness–that is, the non-dual and unconditioned and undifferentiated Brahman–as the fundamental primitive of all existence [6].^d^ Contrary to the idealist, however, mind and consciousness are distinct here. Mind is a feature and a part of the cosmic illusion, ‘Maya’, and that should not be conflated with the only reality, the ultimate Brahman that is ‘Satcitananda’, namely, Consciousness-Existence-Beatitude. While in this mystical philosophy, life, mind, and matter are considered as comprising part of the cosmic manifestation in a world of ignorance, the ’veil of Avidya’, they nevertheless constitute distinct ‘planes’ of existence. The physical plane is one of them but neither the only nor the most fundamental one. This is vividly illustrated in the yoga philosophy that describes a cosmology in which each organism consists of a metaphysical structure made of distinct ‘sheets’ or ‘bodies’: the material sheet (‘annamaya-kosha’), the vital sheet (‘pranamaya-kosha’), the mental sheet (‘manomaya-kosha’), the sheet of divine knowledge or gnosis (‘vijnanamaya-kosha’) and, at the summit, standing above all, the ‘Atman’, the individuation of Satchitananda [7]. Thus, matter, life, and mind are all derivative powers and instruments of fundamental consciousness, not separated and self-existent identities, but nevertheless to be distinguished and realized as distinct from each other. This is reminiscent of the Western Spinozian metaphysical view, in which matter and mind are just two possible ‘modes’ (the ‘res extensa’ and ‘res cogitans’, respectively) of the same fundamental and universal ‘Substance’.

Thus, these metaphysical ontologies do not posit a priori life, mind, and consciousness as emergent properties reducible to material biological processes. They claim that a ‘life-principle’ and/or a ‘mind-principle’ exist that are inherent in organic matter, but distinct from it.

The question this paper pursues is: How far was biology effectively able to dismiss vitalistic and idealistic ontologies? Have these been disproven without appeal, or do these remain a viable option? Though we will point at some findings suggestive of this eventuality, we will not argue in favor of it. Rather, we will argue that this multidimensional conception of life and matter that one finds throughout all cultures and all times, contrary to common belief, has not been dismissed conclusively. We highlight how these conjectures about the nature of life and the mind need a more careful review, especially in light of recent findings.

In the first part, after a short review of the main historical conceptions of vitalism, we will discuss whether its core concepts have been falsified. In the same line of inquiry, in the second part, we will analyze if and how cognition, as well, might be a fundamental principle inherent but distinct from life. Conclusive remarks will follow.

## II. The life in matter

### 1. A short history of vitalism

The history of vitalism, and the meaning attached to it, is long and variegated. We will not go into the details here (for a review, see [8]). It may only be said that, conventionally, one distinguishes between ‘physical vitalism’ (or ‘scientific vitalism’ or ‘process vitalism’ or ‘structural-functional vitalism’) and ‘metaphysical vitalism’ (or ‘essential vitalism’).

Metaphysical vitalism is thought of as a special vital essence, the ‘vis essentialis’ that supposedly infuses and animates all organisms and demarcates living from non-living matter and a ‘vis mediatrix’ responsible for the action and coordination of bodily parts. Already, Plato and Aristotle posited that there is a force immanent to the organisms which supposedly makes life fundamentally distinct from non-life. Aristotle hypothesized that the soul (the psyche) organizes the form and structure of an organism and its purposeful activity. Later, Galen (129–210) assumed that spirit (pneuma) was the essential principle of life, an idea that continued throughout the Middle Ages. It became a matter of scientific debate when French biologist Jean-Baptiste Lamarck (1744–1829), better known for his foundational contributions to evolutionary biology, postulated the existence of an ordering ‘life-power’ augmented by an inner ‘adaptive force’ with which each organism adapts to the environment. In this view, these powers supposedly stand behind evolution and are responsible for the increasing complexity of organisms. Part of this conceptual system was also Lamarck’s famous notion of soft-inheritance (contrasted with the hard-inheritance based on genetic inheritance independent from environmental factors)–that is, the notion that physical traits can be passed on to offspring even if the parent organism acquired them only in its lifetime through its interaction with the environment. The doctrine of essential life humors also found its way through medical practices. Therapeutic bloodletting (bleeding of patients) was thought of as an effective method to release the excess vital fluid considered the culprit behind every ailment and disease. This practice presumably killed more people than it helped and was finally dismissed as pseudo-science at the end of the 19^th^ century.

Physical vitalism, instead, looked for life’s mechanism, action, and system dynamics. Its main representative was Montpellier vitalism, associated with the writings of the 18^th^-century French faculty of medicine at the University of Montpellier, which did not show any signs of metaphysically laden ‘vital forces’ [9]. It expressed a structural-functional form of vitalism with the celebrated image of the bee-swarm: How can a swarm of bees coordinate its behavior as a unique ‘super-organism’?

Physical vitalism accepts physico-chemical determinism, but differs from the physicalist’s viewpoint in that it does not embrace a reductionist approach cutting down everything to physical concepts. Its most notable supporter was French physiologist Claude Bernard (1813–1878), who argued for the irreducible uniqueness of life, claiming it is impossible to see the organism as the sum of its parts, as a reductionist conception would entail. Instead, it must be seen as an integrated and harmonious whole. Bernard posited homeostasis as the foundational principle of life. It might, therefore, not come as a surprise that Bernard contributed most to the eclipse of metaphysical vitalism.

But the distinction between physical vitalism and the classic physical and mechanistic concept of life is, after all, very subtle, if it exists at all. Modern non-linear complex system dynamics theories in developmental biology (‘organicism’) which describe emergent properties of a system that cannot be explained in terms of the properties of its constituents [10] could be labeled a modern form of process-vitalism as well. Deep down, however, it is only a matter of semantics: These theoretical frameworks remain tightly anchored in the orbit of a non-reductive physicalism.

For the remainder of the article, we will focus on metaphysical vitalism and call it just vitalism, if not meant otherwise.

In 1810, Swedish chemist Jöns Jacob Berzelius postulated that organic compounds could be distinguished from inorganic ones if they require living organisms that, through a mysterious vital force, can synthesize them. However, in 1928 German chemist Friedrich Wöhler showed that urea, an organic compound (CO(NH_2_)_2_), could be synthesized in vitro from two inorganic molecules (potassium cyanate and ammonium sulfate), thus disproving Berzeliu’s conjecture.

French chemist Louis Pasteur (1822–1895) kept his speculations less molecular and proposed that a ‘vital action’ makes life inherently different and special compared to non-living matter. Pasteur was inspired by Francesco Redi’s famous experiment that disproved spontaneous generation (maggots are not generated by rotten meat, but, rather, come from fly eggs), taking this as evidence that life must always originate from life. The thesis was that there cannot be, not even in principle, the spontaneous generation of life from non-living matter.

In 1907, Henri Bergson [11] tried to give further credence to vitalism from the philosopher’s perspective. According to Bergson, the principles of Darwinian evolution are not sufficient to explain evolution’s creativity. In life, there must be something more, an ‘élan vital’–a ‘vital impetus’ or ‘vital force’–responsible for the innovative complexity of nature and the morphogenesis of living beings. Bergson maintained that, also, our urges, desires, feelings, and emotions, which impel us to action, come from the same internal creative impulse. It is this self-impelled force within plants, animals, and humans that determines their effort to overcome the inertia of matter. Bergson’s hypothesis was an attempt to find a compromise between a mechanical and finalistic conception, but was met with indifference or ridicule. British biologist Julian Huxley sarcastically rejected Bergson’s idea, comparing the élan vital hypothesis to that of an ‘élan locomotif’ (‘locomotive driving force’) to explain the operation of a railway engine.

A more advanced neo-vitalist version came from the German embryologist Hans Driesch (1867–1941), who theorized the presence of an ‘entelechy’, an intensive, rather than extensive, substance or entity, determining the organic processes and reminiscent of Aristotle’s entelechy–some sort of vital and self-organizing principle which aims to realize a specific design or purpose. Driesch articulated his theory with what he took to be a mindlike essence in all living things, observing the development of sea urchin embryos into whole organisms.

However, despite all the vitalistic concepts that regularly arose throughout history, in the modern scientific context, any form of vitalism has been abandoned as an obsolete working hypothesis. Meanwhile, in contemporary culture, vitalism survives in traditional healing practices, energy therapies, chiropractic therapies, biofield therapies such as Reiki, or other healing methods based on the qi or prana ‘subtle energies’, as postulated in Eastern cultures. It is normally assumed that the former arises as the final winner from the history of science, while the latter are only a form of popular pseudo-science or medieval superstitious healing methods based on spooks, ghosts, and spirits and where a therapeutic effect, if any, can be explained away by placebo effects.

### 2. The meaning of life

The dismissal of vitalism is based mainly on three pillars.

First, a non-material vitalism is metaphysical in the sense that it postulates the existence of a nonphysical ‘life-fluid’ that, per definition, is not detectable by physical means and, thereby, positions itself beyond the boundary of conventional scientific investigation. We defer further discussion of this point to section 6, where we will question if that is really the case.

Second, vitalism is ignored by the adoption of an acritical use of principles of parsimony–namely, Occam’s razor–according to which we should refrain from multiplying entities and posting any hypothesis that is not in line with the dominating paradigm. “Vitalism is an unnecessary hypothesis” is the instinctive objection. But, as we shall see, so far science has not been able to furnish any proof of the sufficiency of the opposite hypothesis either, namely, that life can be reduced to life-less mechanistic processes. While Occam’s razor can offer useful methodological guidance for simplifying our conceptual frameworks and diminishing an experimental workload, it is not a criterion that makes a theory ‘scientific’ or a more complex working hypothesis ‘false’.

Third, the exclusion of any vitalistic hypothesis is based on hope. The hope is that sooner or later everything will be recast in the current paradigm. It is a hope upheld by the belief that it is only a matter of time, funds, and research before everything will be explained inside a naturalistic mechanistic framework. However, facts have shown that the findings of the last century made vitalism even more difficult to expunge. As we will show, the organization and function of life revealed themselves to be much more complex than expected, and the goal of eliminating any vitalistic and teleological temptation appears today to be even further away than it was a century ago. The explanatory gaps did not shrink; rather, they grew.

In fact, the question is: Has vitalism been disproven?

The answer depends on what we mean by vitalism. If we assume that it is a lack of vis essentialis leading to an imbalance of Hippocratic humors (blood, phlegm, black and yellow bile) as a cause of illness and that this imbalance must be reestablished by bloodletting therapies, or that flowing through the nerves is a vis mediatrix that allegedly explains muscle contraction, then, of course, these hypotheses are no longer tenable. Or, if we posit organic vs. non-organic compounds as a demarcation line between living and non-living matter, thereby embracing Berzelius’s criterion and definition for vitalism, then, obviously, with Wöhler’s experiment, the case could be considered closed.^e^

But did science answer the primary and original conjectures of Aristotle and Plato first and, later on, disprove Lamarck’s, Pasteur’s, Bergon’s, or Driesch’s speculations?

Though there is no universally accepted definition, vitalism can be described in its original intent as an answer to the following intuitions.

1. Our inability to explain and define what is life is due to the fact that, in living matter, there is something fundamentally different from what is in non-living matter.
2. The inner psychological force of an organism driven by intentionality, desire, the instinct of survival, striving, will, aim, purpose, and motivations cannot be reduced to mechanistic principles alone.
3. The origin of life is not explicable by the current laws of physics and chemistry alone.
4. The growth and development of biological form cannot be explained by mechanistic principles alone.
5. The creative inventiveness of nature generating all the morphological diversity of species cannot be captured by mechanistic principles alone.

We will argue that, contrary to common belief, these fundamental statements have not been disproven; they were simply ignored. Plato’s and Aristotle’s doubts have not been dispelled. Developmental biology is still far from explaining the growth and development of an organism. The Darwinian paradigm alone, especially in its ‘modern synthesis’ version, turned out to be insufficient to explain the variety and morphological development of species. Vitalism does not deny Darwinian evolution based on principles of natural selection, genetic drifts, random mutations, environmental factors, adaptation, etc. Rather, it finds it unconvincing that these are self-sufficient and fully explanatory principles. That these metaphysical claims were disproven was and remains an assumption unsubstantiated by scientific facts. Science does not know how to explain, in a naturalistic framework, the origin of our instincts, will-force, and cognition, let alone consciousness (the distinction between consciousness and mind or cognition will be clarified later), and is nowhere near able to generate life in a test tube.

### 3. A short reply to J. Huxley

First, let us consider J. Huxley’s counterargument, as it is one of the most cited objections. Huxley misses Bergson’s point; the question is not so much what stands behind the physical motion force of organisms, which obviously can be explained away by mere physical and chemical reactions. A simple electrical impulse can lead to a muscle contraction even in a dead body, as the Italian physicist Alessandro Volta, well known as the inventor of the electric battery, first observed by applying an electric current to the amputated legs of a frog. The question is: Where does the creative impulse of novelty and the urge to reach an ever-increasing complexity and variety of forms we observe in the evolution of life come from? Why does every living being act as if having agency, aim, and purpose if it is a bottom-up construct of aimless and purposeless molecules without agency? And what is and where does that will to survive, will to action, will to reproduce, will to grow, will to expand, will to know come from, making living matter so distinctive from non-living matter? Genetics, biophysics, chemistry, and biology explain the mechanical workings and energetic dynamics of the material substrate, but do not tell us anything about the volitional force, the coming into existence of a conscious subject, the will that determines and moves it. Vitalism does not pretend to explain the nature of the mechanical principles and forces that set an organism in motion. Rather, it posits the existence of an intentional principle, a ‘desire-force’ and a ‘will-force’ inherent to the dynamics of life. Meanwhile, there is no sign that the élan locomotif, namely, the steam’s heat transformed into mechanical energy, imparts to the railway engine any creative force or will to generate new engines or leads it to a novel behavior with agency and purpose other than endlessly spinning the flywheel.

These misinterpretations come from the improper use and interpretations of phrases such as ‘vital force’ and ‘vital energy’. Here, one means, first and foremost, a psychological property or, eventually, a subtle trans-physical ‘substance’ inherent in living matter, but not, or not necessarily, something to relate to the strict notion of force and energy in physics. Whether these vital ‘forces’ or ‘energies’ must be intended literally as physical properties, namely, of the ability to impel a change in motion in time or to produce physical work on material objects, is a secondary matter. The question remains: What makes living matter different from non-living matter and why does a neural network produce life instincts and a psychological ‘will-force’? On the other hand, the misnomers of ‘force’ and ‘energy’, in the context of vitalism, are also not so misplaced either because, as everyone knows all too well, an ‘emotion’, whose etymology comes from the Latin ‘emovere’, meaning to stir or agitate, can be a quite powerful ‘force’ of life that can set us pretty much in motion.

These are details and subtleties that, nonetheless, make a whole difference, all of which Huxley ignored, commenting only on the external material dynamics of a living being, but making little or no connection to its whole internal psychological dimension.

### 4. Genomecentrism, morphogenesis, and synthetic biology

Until recently, biology answered to Bergson’s hypothesis of a ‘creative morphogenesis’ and to Driesch’s ‘vital self-organizing principle’, more elegantly than Huxley, with a naturalistic counter-hypothesis: There is no life force because every morphological organization and its development can be explained in terms of genetic expressions, and their mutation in time is determined by their interaction with the environment.

This was a hypothesis that could, in principle, explain the appearances. The premise was that all our morphological traits are encoded in the DNA molecule, like a ‘book of life’. This was held for a long time as the most plausible working hypothesis. But the data that emerged from the Human Genome Project shortly after the turn of the millennium showed unequivocally how this idea, which was dominant throughout the 20^th^ century, required substantial revision. One of the main findings of the genome mapping and analysis of the past two decades was that the complexity of an organism does not scale with the number of genes in the genome (the ‘C-value enigma’). The DNA’s functionality is different than previously imagined and much more flexible than thought; it is no longer felt by many to be a control kernel of life. It serves mostly as a template for amino acids that make up polypeptide chains transcribed into RNA, then translated into proteins. (In fact, only a small fraction of the genome is actually translated; the rest is ‘non-coding DNA’–the infamous and inappropriately named ‘junk DNA’.) It is not the genotype that determines the phenotype–that is, the DNA does not specify how proteins will have to be assembled to create the anatomical architecture of a fully developed organism. DNA is not a blueprint and does not function like a ‘computer program’ that codes for the morphology of the body (for reviews, see [12], [12, 13], or a summary of these [14,15].)

Meanwhile, the instructions to form an organism arise from an extremely complex network of interactions and relations between components, such as a myriad of cell signaling factors, enzymes, other proteins, amino acids, vitamins, minerals, etc. Genes are a passive database to build proteins, but they neither design nor control the shape, form, and structure of a living being. We now know that the DNA codes for the ingredients not for the recipe. From epigenetics, we now know that heritable phenotype changes are possible without altering the DNA sequence, involving instead the gene activity and expression which, among other things, may result from how the organism responds to external or environmental factors. There are complicated non-genetic factors that cause the organism’s genes to behave differently.

Of course, physical traits like hair, skin, and eye color, facial features, and so on depend on the inherited gene pool. But none of these traits originates from a single gene; rather, they are the result of several streams of chemical synthesis, controlled by regulatory networks whose dynamics govern the genetic transcription. Even this results, in most cases, in a statistical outcome, not a certain and predetermined fact. More evolved functions depend upon even vaster regulatory networks and thousands of genes. Genes are only one of the players in an incredibly complex process inside the whole cell. Moreover, genetically identical individuals do not grow identically, even if subjected to the same environment, while large genetic variations can lead to the same phenotypical trait via multiple alternative pathways [16]. Cells and organisms can also alter their own DNA, rewriting their genome throughout life [17]. By reading the DNA, one cannot determine the shape and size of an organ of a creature. DNA does not even tell us if there is a particular organ at all because it is not the DNA molecule that determines this.

As genomic studies have shown, we humans share 98.9% of our DNA with chimpanzees [18]. The remaining 1.1% codes for olfactory receptors, some having to do with the size of the pelvic arch, which allows us to walk upright, for fur, and differences in the immune system. It is not clear if the cognitive abilities between humans and chimps are a matter of genetic difference. What is known is that the genome determines a cognitive difference because of genes coding for a higher number of rounds of cell division during fetal brain development, leading to thrice as many neurons in the human brain as in that of a chimp brain. The genes encode only for a quantity which, much later, enables a quality, but there is nothing known in the genome that determines that quality. The latter must somehow be acquired with experience by our human neocortex, which embodies the main cerebral differences between our brain and those of other animals. So far, nothing has been found that codes for these skills other than saying “multiply chimp neurons by three”.^f^

Therefore, the popularly conceived genomecentric notion of genes as a blueprint, encoded in the DNA as a program determining the organism’s structure, its development, its variations up to every physical trait, and even our psychological inclinations, does not exist. We are not nearly as determined by our genes as once thought.

Another aspect that turned out to be hard to expunge is the (apparent or real) teleological dimension of life. An intriguing example of this comes from morphogenesis–that is, how, from a single fertilized cell, a highly complex self-assembling pattern emerges, developing the organism appropriate to its species. What are the mechanisms and underlying biophysical and chemical phenomena that preside over complex and apparently goal-driven pattern formations, like the self-assembly of structures such as an eye, a limb, and the entire ‘bodyplan’ during embryogenesis? How do organs regenerate after injury? How can large numbers of cells coordinate their individual activity to assemble themselves into organs and achieve geometric and functional goals that are defined at a macroscopic scale of the whole, but that cannot be found anywhere at the cellular level? Nowadays, we know that there are also correction mechanisms with adaptive decision-making capabilities within living tissues able to correct and adjust embryonic development despite forceful induced defects [19]. An inherent ‘goal anatomy’ that does not stop at the formation of an adult organism, as well-known from the salamander’s amazing ability to regenerate an amputated limb.

Contrary to popular belief, we are far from having any coherent theory capable of explaining how all this works. What we know is that it looks like the self-monitoring and repair of complex multi-tissue organ systems and their pattern formation involves a bioelectric code driving and changing the cell’s transmembrane electric potentials. The large-scale anatomical pattern formation is regulated by very complicated networks of bioelectric signaling among cells which determine the differentiation and regulation of embryonic development and the regeneration of injured tissue and even avoid tumor formation [20].

Science is now trying to reduce these processes to ordinary electro-chemistry by speaking of hugely complex ‘long-range signals’, ‘planar polarity of proteins on cell surfaces’, ‘standing waves of gene expression’, ‘trans-membrane voltage potential and tensile forces’, and ‘chemical morphogen gradients carrying information about both the existing and the future pattern of the organism’ [21]. Some speak of a ‘teleophobia’ that *“has gone too far, putting biologists into a straitjacket that prevents them from exploring the most promising hypotheses”* [22].

Other researchers have placed biological development and morphogenesis in terms of an information architecture. The multicellular organism is considered beyond the material aggregation of material units: It is a whole complexity of a communicative assembly of cells seen as an integrated cellular information field. Individual cellular information fields aggregate into an architectural matrix that enables organism-wide information management [23–26]. From this perspective, multicellularity is collaborative cellular management directed toward the optimization of information quality through its collective (internal and external) measured assessments. The biological organization represents a dual heritable system: It constitutes a biological materiality and also a conjoining information architectural matrix, with morphogenesis deriving from the reciprocations between these two inter-related facets and thereby yielding coordinated multicellular growth and development. Hence, an information architecture could serve as a morphogenetic template. This could be deemed a natural bridge between vitalistic concepts and physical/energetic realities. The logic is that in the cognitive frame, information is physical, not ethereal.

Thus, while previously all hope was laid into a gene-centric approach, and that sooner or later would have abolished any vitalist temptation, now developmental biology resorts to bioelectric molecular signal functions at long range or information-theoretic approaches. To date, however, morphogenesis, despite a century of studies, is to a large degree still shrouded in mystery. The original Aristotelian vitalist claim that the creative generation of the form of living organisms needs a vital force and/or a goal-directed process, and that there must be a ‘mind-like essence’, an indwelling ‘idea-plan’ of assembly, without which life cannot develop, is far from having been expunged.

This also raises the question of whether the generation of life by artificial means would dispel any form of vitalist conceptions. The recent developments in synthetic biology come to mind. Synthetic biology is a rapidly growing field of science that aims to redesign organisms for medical and agricultural applications, employing biotechnology, genetic engineering, molecular biology, and other methods of chemical, biological, or computer engineering. A 2007 Nature editorial declared synthetic biology as providing “a welcome antidote to chronic vitalism” [27]. The claim was that once science became able to synthesize life, such as building protocells from macromolecular constituents, that would finally discard any form of vitalism. This was questioned more recently, showing that a synthetic approach would nonetheless be consistent with a physical–that is, a functional or organizational–form of vitalism [28]. Meanwhile, the question of whether it would dismiss metaphysical vitalism is not that straightforward either. It may only be said that the ability to engineer, design, recreate, or reproduce something does not necessarily imply that one can explain it and understand which forces are, or are not, at work. Also, relatively simple natural phenomena can be reproduced artificially without the ability to explain the mechanisms that make them come into being and which are the underlying forces and efficient causes.^g^

However, for the time being, this would be a purely speculative discussion. While, to a certain extent, synthetic biology made impressive progress, the creation of primitive cells does not go further than the design and synthesis of a minimal bacterial genome [29,30], protometabolic self-replicating molecules [31,32], or primitive membranes, such as lipid vesicles (liposomes), encapsulating macromolecules [33]. But creating the genetic material to enact the molecular machinery for protein synthesis and the metabolic pathways’ complex network biochemical reactions, with all the associated functions performed by the cell´s organelles and substructures (nucleus, ribosomes, mitochondria, cytoskeleton, etc.), and, finally, obtaining a structurally stable protocell able to grow and reproduce, pass on heritable traits to offspring, replace worn parts by repair mechanisms, and respond adequately to environmental changes remains a very distant achievement, if possible at all. Therefore, we feel it premature to speculate on the question of whether synthetic biology repudiates metaphysical vitalism, and leave it to posterity.

### 5. What is life?

The other claim of the vitalist is that there is a fundamental difference between living and non-living matter. However, also, the attempt to abolish this distinction by reducing the former to the latter remains an elusive undertaking.

Famous is Erwin Schrödinger’s question “What is Life?” [34], which precisely tried to address this issue, without any convincing final answer. Biology tried hard to define what should be considered a living organism in naturalistic terms. However, there is no consensus regarding a universal definition of life. Nowadays, biology identifies the ‘living’, loosely speaking, as anything capable of metabolizing, eating, excreting, maintaining homeostasis, growing, adapting and responding to the environment, reproducing, and evolving. NASA adopted Carl Sagan’s original proposal to define life as a “self-sustaining chemical system capable of Darwinian evolution” [35]. But one could present several counterexamples of non-living entities which nevertheless satisfy several of the above criteria.

Viruses have no metabolism and remain inactive without reproducing as long as they do not encounter a host cell. And yet, they are capable of Darwinian evolution. Whether or not a virus is to be considered a life form is a matter of debate.

In a sense, cars ‘eat’, ‘metabolize’, and ‘excrete’, but no one would recognize them as resembling life. One might object that cars do not reproduce. But while reproduction is a common trait among all living beings, it cannot be the decisive aspect individuating life because the opposite does not hold: Living beings can also be infertile or sterile. Moreover, computer programs can simulate ‘artificial organisms’–digital cellular automatons–fitting the above definition, though only at a much lower level of complexity than that of a real living cell. Whether such ‘virtual lifeforms’ could be designated as ‘living organisms’ is highly doubtful.

Thus, any definition of life based on such exclusively material functionalism always fails to capture something of what it is trying to define. For some reason, there is something undefinable, ineffable, and intangible in life, escaping a rigorous and universal scientific definition inside a purely reductionist and physicalist paradigm. The demarcation line between what is living and non-living remains unclear. Why is it so hard to define something whose existence is undeniable and whose distinctiveness so evident?

Because of these philosophical issues, others, such as molecular biologist Andrew Ellington, declared: *“There is a more obvious conclusion to be drawn from our failure to define life: there is no such thing as life. Life is a term for poets, not scientists. There are only replicators with different degrees of complexity. PS: Many of you are closet vitalists”* [36]. Indeed, if something cannot be explained inside a particular normative view, one might legitimately conclude that it is a misplaced abstraction and deny its objective existence in the first place, thereby avoiding the burden of defining or explaining it.^h^

However, one may submit another conclusion to be drawn from the failure to define life: Vitalism is always lurking behind the scenes because, contrary to the accepted common narrative, there has never been any serious refutation of it. In life, we see consciousness, will, desire, motivation, cognition, mind, and goal-driven behaviors. Naturalism’s aim to reduce these psychological traits to functional descriptions of material processes remains unsuccessful.

### 6. The mystery of sleep

The author would like to add a short personal conjecture: To the best of my knowledge, no one has connected vitalism to the function of sleep. Let me outline the rationale behind this.

Science is not driven solely by a purely objective, third-person study of reality, but has always been conditioned by the way we perceive the world from a subjective first-person perspective. Consciousness studies are a paradigmatic example of this. From an exclusive third-person physicalist perspective, consciousness should be considered ‘non-existent’, as we do with vitalism. It is only because of our first-person subjective phenomenal experience that we admit something like consciousness, mind, emotions, feeling, and, more generally, sentience as being a subject worthy of scientific and empiric study. Otherwise, we would not allow these ineffable and undefinable quiddities to be part of scientific analysis.

On the other hand, we do not perceive ‘vital energies’ as being part of our inner first-person experiential realm and, thereby, the analogy may not hold. The question, however, is: Do we really not perceive it? Are our inner emotional sensations, feelings, instincts, desires, and all those drives we would label as ‘vital’ of the same nature as the outer physical sensations of touch, sight, sound, smell, and taste? Does the fact that both manifest inside a bodily boundary prove them to be material?

For example, does that feeling of ‘freshness’, that sensation of having ‘recharged the batteries’ we know from our personal experience after a good sleep, come only from the physical? Conversely, does that perception of a ‘lack of vitality’ we know from sleep deprivation indicate only an organic lack of energy? If so, why can that metabolic deficiency not be replaced by something metabolic, say, by nutritional means, and instead requires an apparently passive function such as sleep? When we do not sleep enough, we increasingly feel a sense of a lack of ‘vitality’, ‘energy-deprivation’, and ‘inertia’ that no substance, food, or metabolic process in a waking state can regenerate; only the sleep cycles can restore this balance.

One of the unresolved issues in modern biology and psychology remains the nature and function of sleep and dreams. Findings suggest that dreams have the functional role of processing our emotional waking-life experiences to avoid an informational and experiential overload that we could otherwise barely handle. Scientists agree that sleep has the purpose of repairing and reorganizing neural pathways, consolidating memories acquired during the waking state, filtering out redundant information, restoring the body and mind, and serving other metabolic, physiological, and psychological functions. As is well known, sleep deprivation leads to severe psychophysiological disorders and, in extreme cases, leads to death. However, there is now a growing consensus that this cannot be the whole story and that the real function and purpose of sleep remain elusive (for areview, see [37-39],).

In fact, we do not know why all the functions of sleep mentioned above could not be performed in a waking state as well. Also puzzling is the evolutionary origin of sleep. From a naturalistic evolutionary perspective, sleep looks like an outsider. According to Neo-Darwinism, everything–including sleep–must have evolved from natural selection and random mutations to allow for the best survival and reproduction chances. But sleep is a risky habit in a prey-predator environment, especially for those that are at the bottom of the food chain. Yet, there is no living organism, from cyanobacteria all the way to humans, that is not subjected to a circadian clock that, in brains, expresses itself as what we call ‘sleep’.

The assumption that identifies sleep uniquely as a cerebral cycle is questioned by findings that show how the waking-sleep activity is not something proper only to organisms with a brain; those without a brain also show waking-sleep cycles. A sleep-like state has been observed in the cnidarian Hydra vulgaris, a small freshwater polyp that has only a primitive nervous organization [40]. It is now known that sleep is also present in animals, such as the jellyfish Cassiopea, which possess neurons organized into a non-centralized nerve system, but still have no brain. Their pulsing behavior, alternated by periods of quiescence at night, is consistent with waking-sleep cycles. When deprived of these quiescence periods, their activity and responsiveness decrease, indicative of a sleep-like state and supporting the hypothesis that sleep arose prior to the emergence of a centralized nervous system [41]. This suggests that sleep serves a more fundamental function than neuronal reorganization, primarily metabolic functions [42].

The question remains, however: Why must this metabolic function occur with an altered mind, body, and consciousness state? The vitalistic hypothesis that sleep may serve as ‘recharging’ the ‘life-sheet’ or ‘vital body’ with its ‘vital energy’ is not falsified.

## III. The mind in life

### 1. Consciousness and mind

Vitalism cannot be abstracted from the so-called ‘hard problem of consciousness’ [43], and that also asks for a satisfying closure of the explanatory gap between neural correlates and the subjective experiential and mental dimension of phenomenal consciousness and our inner subjectivity. Life stands in front of us with its cognitive and conscious dimensions as well. Every form of life, including the most primitive unicellular organism, as we will see, displays different degrees of cognitive behavior.

We previously distinguished between life and mind, with the former being characterized by qualities such as desire, emotions, instinct, strife, or will, while the latter relates to cognition, reason, or intelligence. Let us complement this distinction with a few clarifying remarks on the terminological and qualitative distinction between consciousness and mind as well.

In modern consciousness studies, consciousness is usually regarded as the ability to have any subjective experience, sentience, or perception–what the American philosopher Thomas Nagel called “something it is like to be” [44]. If there is something, someone, or anything it is like to have an experience in and of itself, then one is said to be conscious. Loosely speaking, consciousness is what allows us to be a sentient, an experiencing subject witnessing qualitative phenomenal contents–so-called qualia–such as perceptions, feelings, pain and pleasure, sensory data, the perceptions of qualities like the redness of a rose or the sweetness of sugar, emotions, and mental contents.

But consciousness is also conscious *of* something: the fleeting thoughts, the impression of emotions, the sensory perceptions, the film of life, etc. In particular, it is conscious *of* mental events.

At a higher level, mind is the rational intellect, the thinking reason, that organizes and structures into representations, ideas, concepts, and meanings, that analyzes, reflects, makes more or less abstract logical inferences by analytic and deductive thought processes, etc. Cognition is a process of mind. It is the mental act or process of knowing, understanding, and perceiving through thought, experience, and the senses. As we are going to see in the next section, at a lower level, one speaks of ‘basal cognition’, meaning rudimentary learning and decision-making processes, beginning from the dynamic problem-solving capacities of cellular and subcellular forms [45,46].

Therefore, mind must be kept distinct from consciousness insofar that mind is an instrumental entity of consciousness with the cognitive function of thinking and knowing. Consciousness is distinct in the sense that it apprehends (but is not) mental states such as concepts, ideas, images, imaginations, etc. The distinction is necessary not only for semantic clarity, but also because, in hindsight, it is a common first-person experience: While mental contents such as thoughts and ideas come and go, the conscious individual subject witnessing those impermanent thoughts and ideas does not change its identity. To put it metaphorically: Consciousness is the canvas, mental phenomenality is the painting.

According to scientific materialism, mentality, in its low or high-level cognitive function, must be explained in the frame of a reductionist and physicalist formulation. All those cognitive tasks we characterize as ‘mental’ are considered epiphenomena and emergent properties, resulting from biophysical interactions of networked elementary units, such as molecules or neurons, for adaptation purposes of the organism. Meanwhile, the mind-body problem of the philosophy of mind remains, more than ever, an actual debate failing to find a commonly accepted resolution. Despite the enormous advances in neuroscience and consciousness studies within the last decades, consciousness and mind remain one of the most elusive aspects of reality.

This led to the recent revival of metaphysical ontologies, such as panpsychism or idealistic and panentheistic conceptions of a universal mind. Most notably, panpsychism has been reconsidered in its different forms by modern leading philosophers in the field, like Thomas Nagel [47], Galen Strawson [48], David Chalmers [49], and Philip Goff [50], and on the other side of the spectrum, theories of cosmic consciousness, such as Ithai Shani’s cosmopsychism [51] or Bernardo Kastrup’s analytic idealism [52], to mention only the most noted. These posit not matter, but, rather, consciousness or mind, as fundamental.

## 2. Cellular cognition and consciousness and plant basal cognition revisited

There is now sufficient empiric evidence suggesting how at least forms of ‘basal cognition’ necessitate neither a brain nor a nervous system. Mounting evidence suggests how at least cognition might be a fundamental aspect of life, before the emergence of neural activity, while until recently it was taken for granted that any form of cognition could emerge solely from a brain or that it requires at least a neural substrate.

However, biology is discovering that cells have some primitive ability to learn and associate, resembling conditioned behaviors or changing them by anticipatory skills. Within the turn of the millennium, a renewed interest in this field gained momentum, especially due to the new findings that are transforming our understanding of how mentality emerges in living organisms and even questions the very notion of ‘intelligence’ and ‘mind’ itself.

The most notorious example of cellular intelligence is that of the ‘Physarum polycephalum’, a large amoeba-like slime mold ‘plasmodium’*–*a fungal cytoplasm containing several nuclei, but enclosed in a single membrane – that can be considered a single giant cell. It changes its shape as it crawls in search of food, as a yellow network of tube-like structures that grow a few centimeters per hour, a movement that can be captured via time-lapse recordings. This slime mold has several skills and behavioral patterns that could be labeled ‘proto-intelligent’ and that one would hardly associate with such a primitive creature. For example, it can find the minimum length between two points in a labyrinth [53]. Further research showed that P. polycephalum can minimize the network path and complexity between multiple food sources–it can solve the ‘traveling-salesman problem’, commonly known in both biology and computer architecture [54].

Conditioned behavior was shown as well. When it is exposed to life-threatening electric pulses at constant time intervals, it reduces its speed of growth or stops entirely for a while before starting to grow again. Once conditioned, it learns to anticipate the arrival of the shock if one administers a series of pulses and leaves out the last one [55]. P. polycephalum is also able to adjust to unfavorable circumstances. If one forces it to cross an agar bridge with caffeine or quinine at toxic, but not killing, concentrations, it first slows down but, after repeated attempts, nevertheless crosses the bridge at the same speed in the absence of the irritant substances [56]. It was believed that this was something only neural networks could do: learning to ignore repeated negative stimuli. That is, a learning process of habituation took place.

Another quite surprising behavior was (re)discovered recently in another protozoan. Already in 1906 (for a short historical account, see [57]), the American zoologist, Herbert Spencer Jennings, noted how the ciliate ‘Stentor roeselii’ is capable of escalating actions to avoid an irritant stimulus. One hundred and thirteen years later, Jennings’ observations were confirmed [58]. Indeed, this unicellular organism can change its behavior about how to respond to the environment in an escalation of actions that, to date, represent the most complex behavior known for a single cell.

Empiric evidence suggests that conditioned behavior in other unicellular organisms exists, such as in amoebae. A Spanish group analyzed the motility pattern of the ‘Amoeba proteus’ under the influence of stimuli, consistent with associative conditioned behavior [59].

Similar abilities have been observed in bacteria. Bacteria are considered the most elementary form of life because they are prokaryotic cells. Nevertheless, it has been shown that they can sense the environment, actively move within it, target food, avoid toxic substances, and meaningfully change their swimming direction. Most interesting is their behavior when they come together and form a bacterial community, which shows surprising problem-solving abilities. Bacteria communicate with each other and coordinate gene expression, which determines the collective behavior of the entire community by a ‘quorum sensing’. They can cooperatively self-organize into complex colonies through a communication-based intercellular interplay [60].

This leads to a collaboration that allows the community to achieve a common goal. These communities elaborate functional structures to determine if other microorganisms nearby are threatening their survival and eventually secrete antibiotic compounds toxic to other species except their own, anchor in a place or stay motile, divide for the growth of the community, release spores, etc. This allows them to work together to survive in stressful environments. Analogous to P. polycephalum, bacteria’s collective intelligence becomes evident when they are confronted with relatively complex task-solving problems, such as route-finding in mazes and fractals [61]. Cells were observed in sensing a shortcut using self-generated gradients and selecting a new minimal route (see [62] or for a review of bacteria’s basal cognitive skills, see also [63]).

Whether unicellular forms of life can be considered conscious in the sense of having some form of a sentient experience is something hard to establish given the inherently subjective nature of consciousness. Nevertheless, this evidence of cellular intelligence prompted hypotheses regarding the cellular origin of consciousness as well. Noteworthy is the Cellular Basis of Consciousness (CBC) model, which challenges the assumption that subjective awareness emerged late in evolution [64]. Conscious experience may already appear in cellular form, co-terminus with life. Processes taking place in excitable cellular membranes may lead to a basal form of proto-consciousness with intentional and cognitive capacities [65]. In this theoretical framework, the plasma membrane, together with the nuclear envelope/centrosome/microtubules complex, are seen as the two different ‘nanobrains’ generating a ‘cellular self’ with a purposive agency.^i^ Individual cells maintain their identity by measuring information to sustain a self-referential homeostatic equipoise in reaction to the external environment [24]. A self-referential awareness that *is* self-organization, and in which every cell embodies an elemental cognition. From this perspective, one could conceive of a Cognition-Based-Evolution (CBE), complementing Darwinian natural selection processes, in which receptive intelligent cells measure the environment–that is, assess information–and consequently learn and react to uphold self-identity. In a sense, life could be seen as a ‘continuous measurement of information’. For a thought-provoking summary of the CBC theory, see also [66].

What about multicellular organisms? In this regard, it might be worth noting how there is evidence that non-neuronal multicellular organisms also show forms of intelligence. At the turn of the millennium, terms like ‘plant neurobiology’^j^ emerged, drawing parallels between the complex information processing and signaling system in plants and the animal’s neuronal activity [67,68].

To provide some examples, it was shown that garden pea seedlings (Pisum sativum) change their foraging behavior–their direction of growth–if trained to associate a running fan with a light source, shining an hour after the fan’s operation [69]. Another example that raises important questions, not only about the predictive abilities of plants, but also about how they perceive the environment, was an experiment analyzing the goal-directed movements of the same pea plants, which showed that the climbing plant searching for a support to attach to exhibited an anticipatory prehensile mechanism, which gave it the ability to plan its movements before having any physical contact with the support [70]. There is now an extended literature that, especially in the last decade, has consistently shown how plants change behavior and adapt, respond predictively, possess some form of memory, resort to an air and underground communication system based on chemicals, have visual and acoustic signals, have learning abilities and can evaluate their surroundings, make decisions, and even have a social life cooperating with one another (for a not-too-long review, see [71].)

The overall picture that arises from these findings is that, among living beings without a brain, an intimation of collective intelligence arises when several units or ‘nodes’–unicellular organisms–connect and form an informational network. Once several of these nodes signal to each other in a complex connectome, a new order and level of functional skills arise that the single cell does not have. The single cell already seems to have some elementary cognition (and, perhaps, a proto-consciousness), but something new and qualitatively different emerges once these little entities connect in a complicated communication web.

## 3. What is cognition?

These recent discoveries on monocellular or multicellular behaviors raise questions. Is a tiny unicellular creature, which swims through a fluid, hunts for its prey, avoids obstacles, has a memory, and can even predict events in advance, just a piece of complex biophysical machinery? Or should we ascribe to it, at least, some form of ‘basal cognition’ and eventually even some elementary form of sentience? Is a climbing plant that nervously flutters its tendrils throughout space, analyzes the environment, grows toward a support it apparently ‘sees’, and begins to grab for it before even touching it only a machine driven by a chemical reaction, or something to which we could ascribe a form of conscious cognition?

As with the notion of life, the unambiguous definition of what is commonly called ‘cognition’ remains a matter of considerable debate (for a couple of recent works on this matter, see [72] and [73].) Broadly speaking, we can maintain an understanding of cognition as the action or faculty of learning, decision making, sensing and responding, communicating, information processing, and having memory, agency, and associative skills, including high-level forms of cognition that result in analytic thinking and reason, known as the ‘mind’.

A first standpoint, that of a physicalist approach, is that which does not consider the above described cellular behavioral phenomena as proof of what is commonly meant by ‘cognition’, ‘intelligence’, or even less a ‘mind’ or ‘consciousness’. One might regard such a standpoint as an attempt to anthropomorphize non-human behavior and, instead, express cognitive functions in organisms without a brain in terms of system theory, reducing all complex systems dynamics to mere elementary processes: as an emergent property instantiated as an adaptive behavior of a complex nonlinear dynamical system, which rearranges its internal state in response to the stimuli of an external environment. For example, the system theory of autopoiesis, by Chilean biologists Humberto Maturana and Francisco Varela [74], refers to the process of self-creation and self-preservation of living systems, where cognition is seen as a self-referencing mechanism determined by a structural coupling to the environment. It works along the line of Gilles Deleuze, who considered the tendency of life to move toward a greater self-organizing complexity that maximizes difference (the genesis and process of ongoing biological differentiation), with its dynamic potentiality to develop beyond its actuality (life’s tendency to build new organic structures and properties that allow it to perform new functions), and renamed it ‘vitality’, a form of physical vitalism ([75,76],).

The second standpoint, that of a metaphysical approach, is the idea of matter having in itself, a priori, mental or experiential properties; this was considered only by some philosophers, like Gottfried Wilhelm Leibniz, Baruch Spinoza, Alfred North Whitehead, and William James, who expressed a panpsychist view, namely, that everything is fundamentally a form of consciousness or mind. But these speculations did not have, until recently, much influence on the overall established naturalistic paradigm. The science of biosemiotics, a branch of biology that interprets living processes as the production and interpretation of signs, codes, information, meanings, habit formation, and their communication in the biological realm (for an introductory essay, see [77]), came somewhat closer to the idea of cognition as a fundamental aspect of reality, but refrains from making the decisive step toward the reversion of the naturalistic paradigm.

The (more or less implicit) premise of present science is the physicalist and reductionist view in which we must recast everything into a causational chain determined by a system of processes that lead to cognition as an emergent secondary epiphenomenon–that is, something that works by a unidirectional bottom-up process, such as: aggregation of microscopic fundamental interacting units into large-scale network processes → information/code/semantics → cognition. The plausibility of the hypothesis that goes the other way around is rarely addressed (for an interesting exception, see [78]), namely, that cognition is fundamental, and even more fundamental than matter, and that the causational chain works as: cognition → semantics/code/information **→** aggregation of microscopic fundamental interacting units into large-scale network processes.

The observational data that suggest cognitive properties in non-neuronal tissue allow us to posit that cognition might not be just an emergent behavior determined by a complex network of processing units, but, rather, that it is an inherent and basic feature of living matter itself. There might exist not only a life-principle, but also a mind-principle, both expressing a vital-mental polarity.

## IV. Conclusion

We presented some arguments and scientific evidence aimed at critically reviewing the widely held belief that vitalism is dead, with mentality and consciousness having no place in Nature other than being emergent epiphenomenon. This belief holds fast to the prophecy of Francis Crick: *“And so to those of you who may be vitalists I would make this prophecy: what everyone believed yesterday, and you believe today, only cranks will believe tomorrow”* [79].

Upon closer inspection, however, one realizes that there is no end in sight for the search for a mechanistic explanation that reduces life to non-life and dismisses Bergson’s ‘creative evolution’ or Driesch’s entelechy as self-organizing principles, furnishing a naturalistic foundation of the volitional and cognitive behavior in living organisms. For the same reason, modern findings on the relatively complex cognitive behavior of simple life forms without a nervous system also shift us away from a naturalistic reduction of mental phenomena. Life, mind, and consciousness are far from having been naturalized, even from a philosophical perspective. Whatever the truth, as of the date of this writing, their naturalization cannot be considered a closed case. Metaphysical vitalism and idealism remain viable options. Nothing in science prevents us from seeing will, cognition, and sentience as powers of a subconscious Nature trying to express itself in different biological forms. A theoretical framework is possible that sees life and mind as the two aspects of a conscious universe and as two fundamental inherent properties that emerge in and through matter rather than by matter alone. Life and mind, as two distinct aspects of reality, both having consciousness as their origin and matter as their supporting basis.

Moreover, the trouble with expunging teleological temptations might well be due to humans’ tendency to anthropomorphize Nature, as Ellington implied, but nothing prevents us from recognizing life with its impulsive dimension (will, desire, emotions, motivations, etc.), mind with its cognitive dimension (learning, problem-solving and decision-making processes, information and thought processes, reason, intelligence, knowing), and consciousness with its sentient dimension (experience, sensations, subjectivity) as fundamental principles that are already inherent in Nature and that impel the organization and function of living matter itself, rather than being mechanistic emergent appearances alone. Denying these features of the natural world as being exclusive to humans could be seen as a form of anthropocentrism as well.

The dismissal of these paradigms might have been too premature, and they may soon find their way back into our world-constructs, at least in matters of philosophical interest in biology, and, like idealism, panpsychism, and various conceptions of universal consciousness, are already doing in the philosophy of mind. The phenomenon of life emerging from non-living matter, and that of mind from non-mental stuff, is no less perplexing than that of consciousness emerging from non-conscious matter. There is not only a hard problem of consciousness, but also a hard problem of life, and a hard problem of mind.

Seeing life, mind, and consciousness as appearance arising out of a mechanistic clash of material forces and particles, or, on the contrary, as fundamental properties already inherent in matter itself, is not just a philosophical musing, but something that may influence our way of seeing biology and determine the very practical aspects of science and its future progress or stagnation.

## Notes

a In the literature, the terms ‘physicalism’, ‘material monism’, and ‘materialism’ are frequently interchanged. The terms ‘material monism’ or ‘materialism’ were used more frequently by authors of the past centuries. However, nowadays, ‘physicalism’ is a more rigorous term inasmuch as modern physics describes more than just matter, including energy, force fields and physical laws.

b To be distinguished from ‘epistemological’ or ‘subjective’ idealism, which we will not consider here.

c This is a conflation we will later avoid in this paper, in section II.1. For the time being, we maintain the word ‘mental’ as something relating to ‘cognition’, ‘reason’, or ‘intelligence’, but not ‘consciousness’, ‘sentience’, ‘emotions’, or physical sensations.

d The ontologic framework of a fundamental non-material non-dual awareness as foundational to a self-organizing universe has also received some attention in the biological sciences, e.g [**80**].

e In the present context, the word ‘organic’ is misleading. In chemistry, organic compounds are no longer defined as chemicals synthesized in living organisms. An organic compound is any molecule containing carbon-hydrogen bonds. Also, methane is an organic compound and no one would consider it a ‘living organism’.

f But does size really matter? Not really, as the brain of an elephant has three times the number of neurons that humans have, and the weight and volume of a sperm whale brain measures six times as much. The neuroanatomical uniqueness of humans is in the number of neurons in the cerebral cortex: about 16.3 billion neurons vs. the 9 billion within a gorilla and the 6 billion within chimpanzees.

g For example, one can produce electric currents without even suspecting the existence of electron flows and electric fields, or one can reproduce chemical reactions without knowing anything about the laws of atomic physics and quantum mechanics determining it–let alone understand how, deep down, these necessitate the existence of nuclear forces as well.

h This approach is reminiscent of the doctrine of eliminative materialism in the philosophy of mind. Because phenomenal consciousness refuses to be cast into a strict naturalist account, the eliminativist contends that our subjective experiences, such as pleasure and pain, joy and grief, and the existence of our mental states, are just inexistent ‘illusions’.

i Also others, independently from the CBC model, point out how mitochondria already exhibit social behavior by communicating with each other and with the cell nucleus, exhibiting group formation and interdependence, synchronizing their behaviors, and engaging in functional specialization [81].

j Speaking of ‘plant neurobiology’ for an organism without neurons is an obvious misnomer. This, however, has become a common nomenclature.
